# Correlations among Firing Rates of Tactile, Thermal, Gustatory, Olfactory, and Auditory Sensations Mimicked by Artificial Hybrid Fluid (HF) Rubber Mechanoreceptors

**DOI:** 10.3390/s23104593

**Published:** 2023-05-09

**Authors:** Kunio Shimada

**Affiliations:** Faculty of Symbiotic Systems Sciences, Fukushima University, 1 Kanayagawa, Fukushima 960-1296, Japan; shimadakun@sss.fukushima-u.ac.jp; Tel.: +81-24-548-5214

**Keywords:** mechanoreceptor, tactile sensation, thermal sensation, gustation, olfaction, auditory sensation, firing rate, electrochemical impedance spectroscopy (EIS)

## Abstract

In order to advance the development of sensors fabricated with monofunctional sensation systems capable of a versatile response to tactile, thermal, gustatory, olfactory, and auditory sensations, mechanoreceptors fabricated as a single platform with an electric circuit require investigation. In addition, it is essential to resolve the complicated structure of the sensor. In order to realize the single platform, our proposed hybrid fluid (HF) rubber mechanoreceptors of free nerve endings, Merkel cells, Krause end bulbs, Meissner corpuscles, Ruffini endings, and Pacinian corpuscles mimicking the bio-inspired five senses are useful enough to facilitate the fabrication process for the resolution of the complicated structure. This study used electrochemical impedance spectroscopy (EIS) to elucidate the intrinsic structure of the single platform and the physical mechanisms of the firing rate such as slow adaption (SA) and fast adaption (FA), which were induced from the structure and involved the capacitance, inductance, reactance, etc. of the HF rubber mechanoreceptors. In addition, the relations among the firing rates of the various sensations were clarified. The adaption of the firing rate in the thermal sensation is the opposite of that in the tactile sensation. The firing rates in the gustation, olfaction, and auditory sensations at frequencies of less than 1 kHz have the same adaption as in the tactile sensation. The present findings are useful not only in the field of neurophysiology, to research the biochemical reactions of neurons and brain perceptions of stimuli, but also in the field of sensors, to advance salient developments in sensors mimicking bio-inspired sensations.

## 1. Introduction

Current challenges in the development of integrated sensor systems responsive to multiple stimuli such as the force sensation caused by stress or strain [1,2], the acceleration sensation evoked by posture, the thermal or chemical sensation [3], or other sensations induced by humidity, etc., have been put forth as critical issues [4,5]. The common requirements in the design of multifunctional sensors are twofold: a single sensing mechanism and integrated fabrication, both responsive to a variety of sensations. The former has limits to its applicability in multifunctional sensing, while the latter has the problem of the complicated structures that arise during its intricate fabrication process. These problems must be resolved in order to promote useful sensory applications of these sensors in robotics, health care, wearable electronics, etc.

One way to make a breakthrough against these problems is through the fabrication of monofunctional sensation systems responsible for bio-inspired multifunctioning with a single electronic platform. If a single electronic platform with versatile sensing can be made viable, the simplified production process is expected to induce remarkable development in the area of artificial sensors. In addition, a fabricated system that mimics the five human senses in the form of mechanoreceptors, thermoreceptors, etc. is a useful aid [1,2,3,6]. It is important, however, to avoid the creation of structures that are likely to be complex.

Our proposed fabrication technique for artificial sensors mimicking the morphological paradigm of the five senses is expected to be so effective that a single configuration made using our method of electrolytic polymerization can have multifunctional sensing utilizing a state-of-the art magnetically responsive, intelligent hybrid fluid (HF) [7]. The configuration morphologically mimics mechanoreceptors such as free nerve endings, Merkel cells, Krause end bulbs, Meissner corpuscles, Ruffini endings, and Pacinian corpuscles [8] to provide tactile, thermal, gustatory, olfactory, and auditory sensations. Furthermore, these artificial sensors must also be stretchable and flexible so that they can be used in applications in which they endure sustained large deformations, such as artificial skin, wearable human-machine interfaces, applications in rehabilitation, etc. [9,10,11,12,13]. Our proposed artificial sensor made of HF and soft rubber fulfils these requirements. The present article reports multifunctional sensing on a single electronic platform of HF rubber mechanoreceptors, providing adequate firing rates.

Regarding living creatures in biomedical fields, the firing rate is the potential generated by extraneous stimuli and categorized as slow adaption (SA) or fast adaption (FA) so that it may be evaluated not as a multitude of sensations but as a frequency of response [14,15]. It is such a significant parameter as to affect the biochemical reaction of neurons. Research has been conducted predominantly on the perception of stimuli by the brain in the field of neurophysiology. Therefore, it can easily be suggested that the firing rates of the five senses that correspond to the tactile [16,17,18], thermal [19], gustatory, olfactory [20], and auditory [21] sensations show certain correlations, which is a common finding in neurophysiology. For example, the correlation of the firing rates between the olfactory and gustatory sensations is well known [22,23].

HF rubber mechanoreceptors are also expected to facilitate evaluation of the correlations among the five senses utilizing the firing rate. As for the tactile sensation, we clarified in our previous report that SA and FA are based on ion channel systems with electric circuits in HF rubber mechanoreceptors [24]. The morphology of the electric circuit can be evaluated using electrochemical impedance spectroscopy (EIS) [25], which measures alternating current (AC) and is so effective that it is often applied to solid electrolytes involving ionic conductors [26,27,28] and lithium-ion batteries [25]. Thus, EIS makes it possible to determine the firing rates of various mechanoreceptors. Being able to elucidate the correlations of the firing rates of these mechanoreceptors by evaluating the EIS has made it possible to develop bio-inspired mimicking sensors with single electronic platforms for the sensations that correspond to the five human senses.

## 2. Materials and Methods

### 2.1. Materials

**Figure A1:**
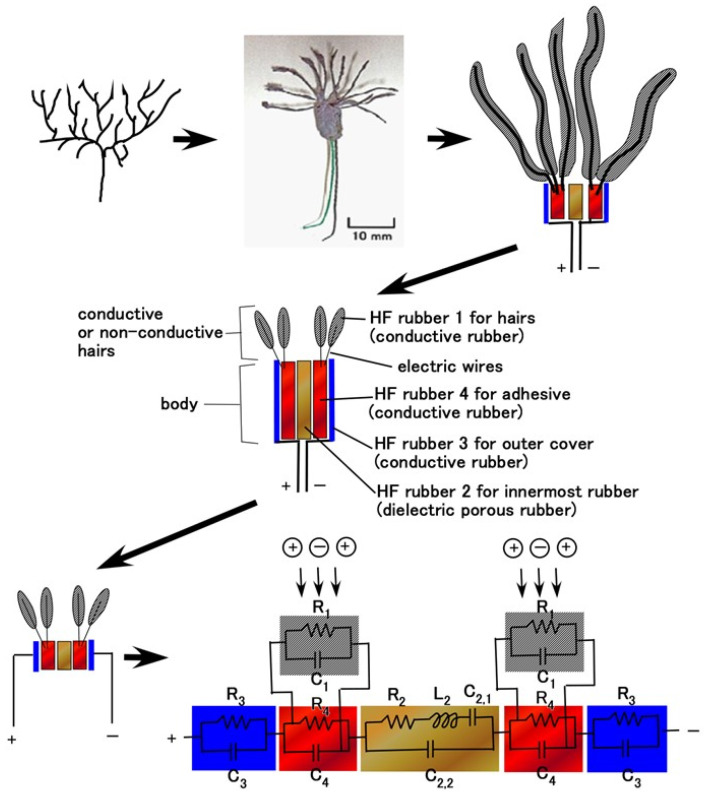
A schematic of our mechanoreceptor. Following the arrows, a schematic of a virtual biomedical mechanoreceptor, an image of the mechanoreceptor fabricated with HF rubber, a schematic of the intrinsic fabrication, the electric structure, the equivalent electric circuit, and an illustration of free nerve endings (Type A) [24].

The present study examines mechanoreceptors with free nerve endings (Type A), Krause end bulbs (Type B), Meissner corpuscles (Type C), Pacinian corpuscles (Type D), and Ruffini endings (Type E). Regarding the tactile, thermal, and auditory sensations, these mechanoreceptors did not have the thin wires protruding from HF rubber 4 shown in Figure A1 in the Appendix A; rather, the wires were embedded in a thumb-shaped molded finger made of soft urethane rubber (U; human skin gel, 0-solidity; Exseal Co., Ltd., Gihu, Japan) whose detailed production process was described in our previous study [7] (the other figures in [7], except for the figures in the Appendix A, aid the readers to understand the contents). The fabricated finger was coated with a combination of natural rubber (NR; Ulacol; Rejitex Co., Ltd., Atsugi, Japan) and chloroprene rubber (CR; 671A; Showa Denko Co., Ltd., Tokyo, Japan). For the gustatory and olfactory sensations, the mechanoreceptors had thin wires protruding from HF rubber 4 and were used in a bare configuration rather than embedded in a finger because bare, thin wires are better able to capture the ions and electrons in the liquids and odors.

Regarding the fabrication process, briefly, the mechanoreceptors were produced using our proposed novel rubber-solidification technique of electrolytic polymerization on the rubber mixing HF, NR, CR, and mm-order Ni particles, which allows for a relatively simple production process. In addition, electrolytic polymerization in conjunction with compounding a metallic hydrate such as Na_2_WO_4_·2H_2_O produces a rubber that is porous and includes the conjugation between the rubber and the metal of the electric wires from which the electric signal is extracted. HF contains water, kerosene, silicon oil, polyvinyl alcohol (PVA), Fe_3_O_4_ particles, Fe particles approximately 50 μm in size, and sodium hexadecyl sulfate aqueous solution for the surfactant; it is thus an optimal fluid for electrolytic polymerization. The electrolytic polymerization is conducted under the application of a magnetic field, so that magnetic clusters are structured by the metal particles to be aligned along the direction of the applied magnetic field, which alignment induces the enhancement and anisotropy of the conductivity.

### 2.2. Methods

The experimental apparatus used to obtain the characteristics of the tactile, thermal, olfactory, gustatory, and auditory sensations is the same as that described in our previous studies [7,24]. The procedure is summarized as follows.

**Figure A2:**
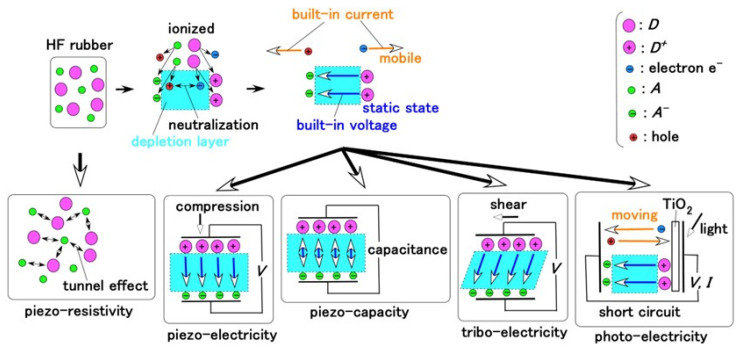
Intrinsic conditions of the HF rubber [7].

Regarding the tactile response to mechanical stimuli, a normal force was applied to the mechanical apparatus with an up-and-down motion continuously a few times, touching the bottom of the vessel at a velocity of 300 mm/min. The finger with the embedded HF rubber mechanoreceptor was moved in hot or cold water and the voltage was measured. The voltage induced in the HF rubber mechanoreceptor results from the built-in voltage, as shown in Figure A2 in the Appendix A.

Regarding the thermal sensation, the finger embedded with each HF rubber mechanoreceptor was pushed once onto a heater in the atmosphere by compression at a velocity of 300 mm/min and the voltage from the finger was measured.

Regarding gustation, a bare HF rubber mechanoreceptor that was not embedded in the finger was touched to the test liquid at a velocity of 100 mm/min and the voltage from the receptor was measured. Cyclic voltammogram plots of the relationship between the electric current *I* and voltage *V* measured with a potentiostat at a 50 mHz scan rate with a potential domain of −1.5–1.5 V were also generated. The liquids used for this test had several concentrations with five different taste sensations: sweetness (a sugar solution in water), saltiness (a salt solution in water), sourness (a rice vinegar solution in water), bitterness (a familiar coffee solution in water), and umami (a Japanese tuna powder solution in water). The redox potential (ORP) and pH of the liquids were measured.

Regarding olfaction, a bare HF rubber mechanoreceptor was inserted into a container filled with 28 wt% ammonia gas and the voltage from the receptor was measured. We also measured the relation between *I* and *V* using a potentiostat under the same conditions as those used for gustation.

Regarding the auditory sensation, the finger embedded with each HF rubber mechanoreceptor was pushed on a soft membrane with a 3619 GPa Young’s modulus attached to a speaker. When a sound was played, the voltage from the finger was measured.

Finally, the AC electric properties of the HF rubber mechanoreceptors were measured using an inductance/capacitance/resistance (LCR) meter (IM3536; Hioki Co., Ltd., Ueda, Japan). Their impedance, etc. were evaluated based on the behavior of the resistance in the AC circuits, in which a time-varying current was generated.

## 3. Results and Discussion

### 3.1. AC Electric Properties

**Figure 1:**
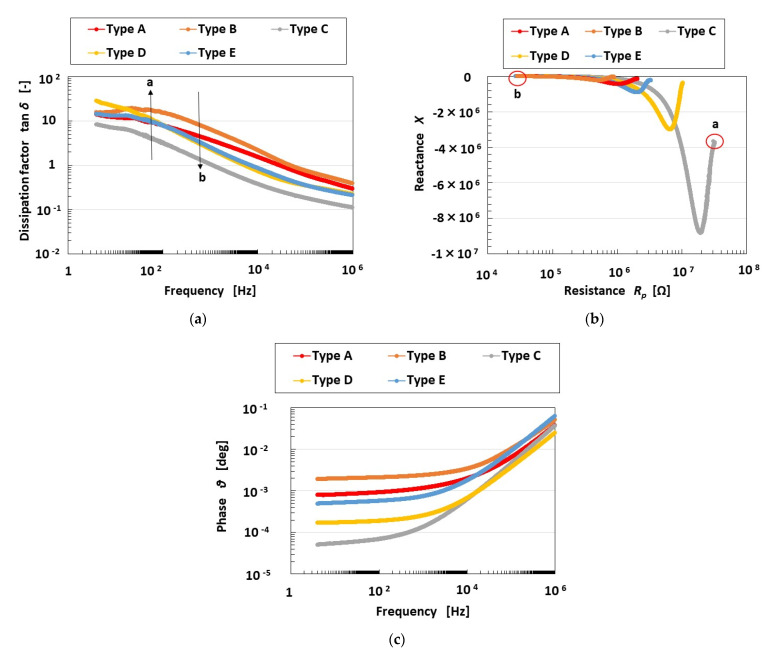
EIS results of HF rubber mechanoreceptors: (**a**) dissipation factor; (**b**) relation between reactance and resistance; (**c**) phase.

**Figure A3:**
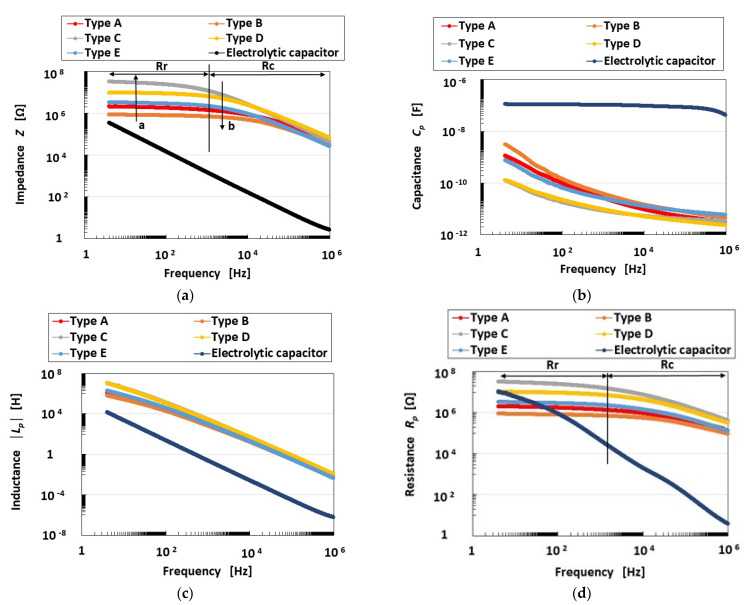
EIS results of the HF rubber mechanoreceptors: (**a**) impedance; (**b**) capacitance; (**c**) inductance; (**d**) resistance [24].

The measurement results are shown in Figure A3 in the Appendix A regarding impedance *Z*, capacitance *C_p_*, inductance *L_p_*, and resistance *R_p_*, and in Figure 1 regarding dissipation factor *tan d* and the relation between reactance *X* and *R_p_*. The EIS is able to deduce the results of equivalent electric circuit models such as other artificial skin sensors [29]. In general, the relation between *X* and *R_p_* corresponds to a Cole–Cole plot or Nyquist plot generated by the EIS results. It is well known in the field of solid or ionic conductors that the intrinsic configuration of a substance can be identified from EIS results.

**Figure 2:**
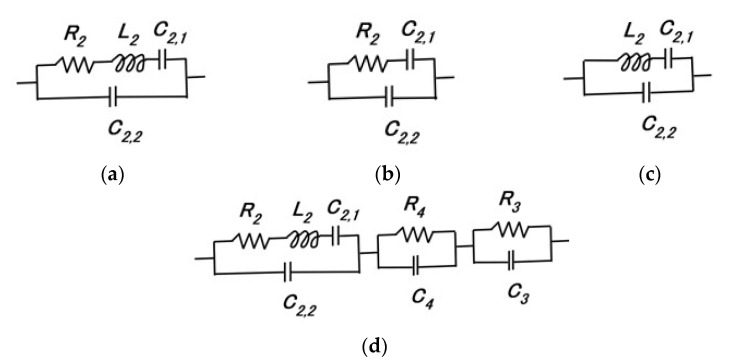
Electric circuits of HF rubber mechanoreceptors: (**a**) approximated parallel circuit of *R_2_*, *L_2_*, *C_2,1_*, and *C_2,2_*; (**b**) approximated parallel circuit of *R_2_*, *C_2,1_*, and *C_2,2_*; (**c**) approximated parallel circuit of *L_2_*, *C_2,1_*, and *C_2,2_*; (**d**) primary electric circuit of HF rubber mechanoreceptors.

**Figure 3:**
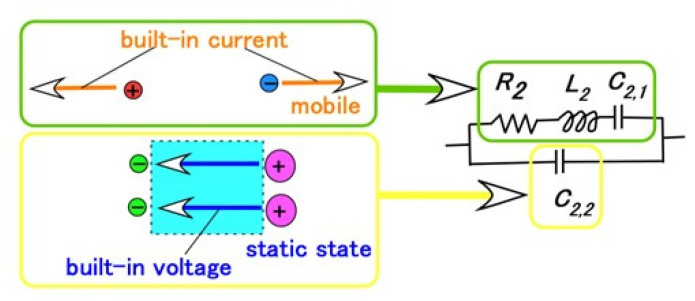
Relation between the behavior of ions, particles and electrons, and the electric circuit.

Taking into consideration the intrinsic structure demonstrating the behavior of the electrons, the electron hole, and Types A and D in the HF rubber as shown in Figure A2, the primary electric circuit can be considered to have the format shown in Figure 2d. It was demonstrated in our previous study [24] that ionic polarization [30,31] is dominant in HF rubber. The unit of *R_2_*, *L_2_*, *C_2,1_*, and *C_2,2_* presents the electric circuit of HF rubber 2, as shown in Figure 3: the integrated impedance of *R_2_*, *L_2_*, and *C_2,1_* is related to the built-in current corresponding to the bulk resistance (or charge transfer resistance) induced by ionic and electronic migration, which appears as ionic and electronic conductivity in the field of solid electrolytes [32,33]. The *C_2,2_* capacitor is related to the built-in voltage corresponding to the double-layer capacitance in the field of solid electrolytes [32,33]. Resistance is very small between the electrode and electrolyte of the HF rubber corresponding to *R_4_* and *C_4_*, which forms the electric circuit of HF rubber 4, because one more loose peak does not exist at the low frequency range, as shown on the right side of point “a” in Figure 1b. The resistance in HF rubber 3 and in other parts of the electric circuit of the HF rubber sensor is very small because one more loose peak does not exist at the high frequency range, as shown on the left side of point “b” in Figure 1b. Thus, the electric circuit shown in Figure 2d is the result of the *R_2_*, *L_2_*, *C_2,1_*, and *C_2,2_* configuration shown in Figure 2a, and the HF rubber 2 is dominant.

Consequently, the HF rubber mechanoreceptor has the same morphological electric circuit, shown in Figure 2a, as ultimately resulting from that shown in Figure A3 in the Appendix A, which appeared in our previous study [24]. HF rubber 2 comes to be a condenser, as do the dielectric materials, including *R_p_*, *C_p_*, and *L_p_*, and the HF rubbers 1, 3, and 4 conductive materials, including *R_p_* and *C_p_*. We illustrate Figure A3 in the present paper as just one schematic of a mechanoreceptor; other HF rubber mechanoreceptors were presented in our previous study [24]. HF rubber 2 is a permeable rubber that can be involved with any liquids because it is porous. In the present study, glycerin was included in the rubber so that the rubber would be a capacitor. HF rubber 3 was used for the outer cover of the sensor, and HF rubber 4 served as an adhesive between HF rubbers 2 and 3 and between HF rubber 4 and the electric wire. HF rubbers 3 and 4 are conductive.

Thus, the consequential equivalent electric circuit as shown in Figure 2a can be seen from Figure 1b. The quantitative tendency of the relation between *X* and *R_p_* to have a loose curve with one peak and a negative value of *X* indicates that the electric circuit is parallel, with the integrated impedance of *R_2_*, *L_2_*, and *C_2,1_* and the capacitor of *C_2,2_*, as shown in Figure 2a. The result, that the HF rubber mechanoreceptor demonstrates the structure shown in Figure 2a, can also be obtained by the result of Figure 1c that phase *θ* is negative throughout the frequency range.

In Figure 1a, the larger *tan d* values (designated as “a”) indicate that the resistor is dominant over the inductor in the electric circuit; this is illustrated in a morphological electric circuit in Figure 2b. The changing tendency (designated as “b” in Figure 1a) is shown in Figure 2c. Therefore, in Types C, D, E, A, and B, in that order, the inductor incrementally dominates.

*X* includes both inductive reactance and capacitive reactance. Then, a comparison between the multitude of values of capacitance *C_2,1_* and *C_2,2_*, and that of inductance *L_2_*, demonstrates that the capacitance is larger than the inductance, given the negative values of *X*. However, as shown in our previous study [24], owing to the existence of the inductance, the firing rate is FA. Additionally, the larger absolute values of *X* indicate that the inductor dominates in the electric circuit; therefore, in Types C, D, E, A, and B, in that order, FA is incrementally demonstrated.

Furthermore, “a” in Figure 1b in the region of larger *R_p_* values denotes a smaller frequency range, with the result that the right side of the abscissa in the figure indicates a smaller frequency range. Conversely, “b” in Figure 1b denotes a higher frequency range. The absolute value of *X* at the lower frequency range (“a”) is larger than that at the higher frequency range (“b”). At the higher frequency range, the parallel electric circuit is dominated by C*_2,2_* (Figure 2a), whose absolute *X* value is small, while at the lower frequency range it is dominated by the integrated impedance of *R_2_*, *L_2_*, and *C_2,1_*, whose absolute *X* value is larger than that at “b”. Therefore, C*_2,2_* is smaller than the integrated impedance of *R_2_*, *L_2_*, and *C_2,1_*.

### 3.2. Firing Rate

**Figure 4:**
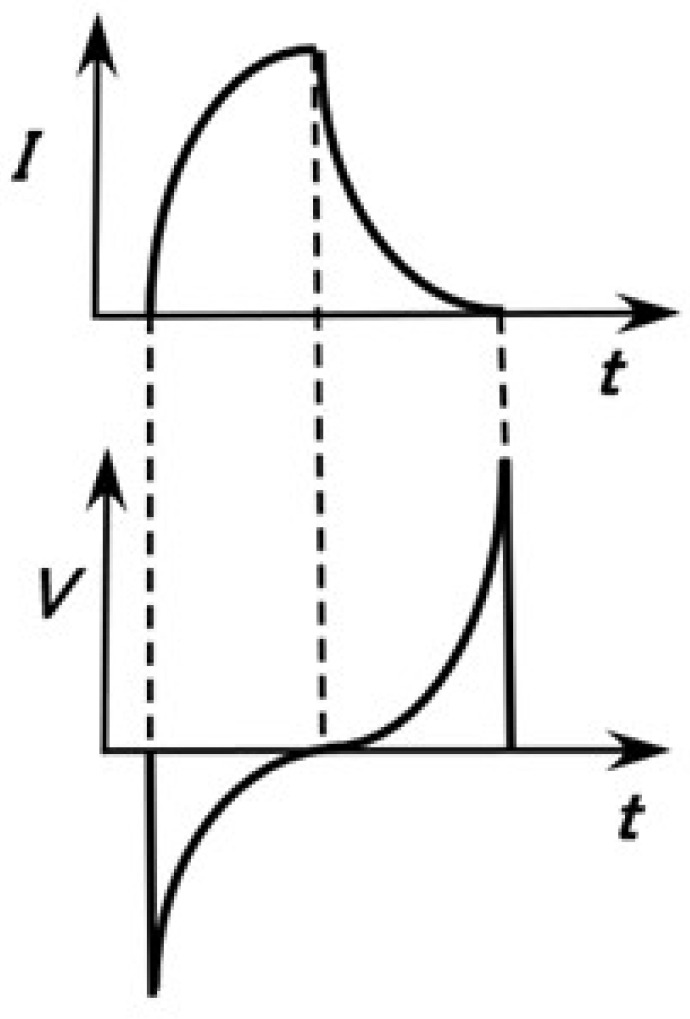
Relation between the voltage and the electric current in the inductor.

In the time that the skin takes to respond to extraneous stimuli, the mechanoreceptors generate voltage changes as electric impulses. These changes are manifested as the firing rate, which is based on the frequency of the electric impulses. The electric impulses present as the electric current that runs through the neuron created by the action of the electrons, electron holes, and ions in the human body. Therefore, the rate of the electric impulses can be estimated based on the electric current in an ionic conductor such as an HR rubber mechanoreceptor. On the other hand, the morphological structures of ordinary inductors are categorized as multi-layered, spiral, membranous, etc., and their configurations are similar to those of the HF rubber sensors fabricated with HF rubbers 2–4. Therefore, any given HF rubber sensor corresponds to an inductor as shown in Equation (1), with inductance *L*, where *t* is the time. Figure 4 shows *V*, taking into consideration changes in the electric current *I* in the inductor (Figure 4 and Equation (1)). Abrupt changes in *V,* such as sharp peaks, denote the firing rate. Therefore, changes in inductance *L* and reactance *X* involving inductive reactance are related to the firing rate, and the firing rate can be determined by evaluating *L*, *X*, etc. measured by EIS.

(1) $$V=L\frac{dI}{dt}$$

In addition, as discussed in our previous study [24], the firing rate is the differentiation of the mean spike count of *V*, as shown in Equation (2), where λ(*t*) is the firing rate, *N* (*t*) is the mean spike count, and ε is the diminutive increment of *t*. Thus, the firing rate can be estimated as the gradient of the changing voltage.

(2) $$\lambda \left(t\right)=\underset{\varepsilon \to 0}{\lim}\frac{N\left(t,t+\varepsilon \right)}{\varepsilon }$$

**Figure A4:**
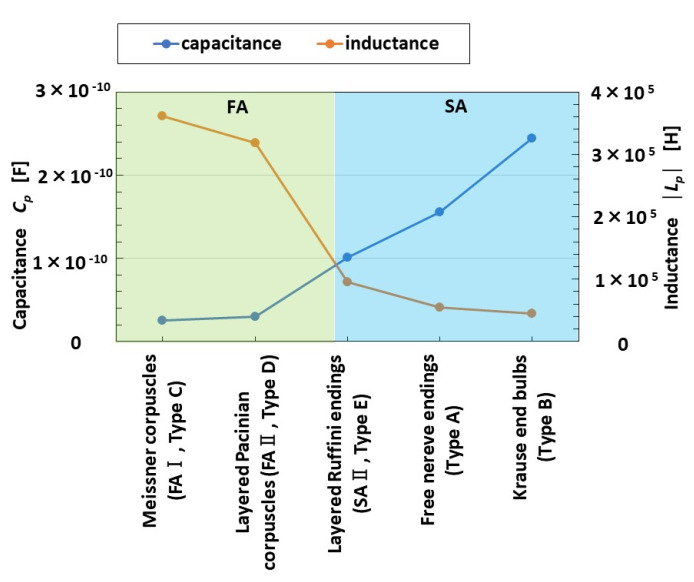
Relations among the fabricated mechanoreceptors, capacitance, and inductance [24].

Regarding the tactile sensation, the equivalent firing rate can be obtained as presented in our previous study [24]: Types C and D show sharp peaks, and their equivalent firing rates change abruptly and correspond to FA; Types A, B, and E show multiple peaks with the same quantitative magnitude and have equivalent firing rates without sharpness that correspond to SA. The relations among the firing rate, *C_p_*, and *L_p_* can be summarized as shown in Figure A4 in the Appendix A.

**Figure 5:**
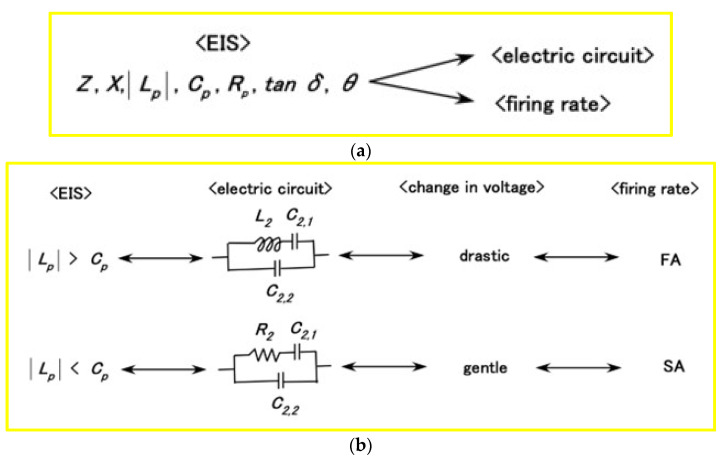
Illustration summarizing the relations: (**a**) among EIS, the electric circuit of the substance, and the firing rate; (**b**) among EIS, the electric circuit of the substance, the changes in voltage of the sensor, and the firing rate on FA and SA, respectively.

Incidentally, we can provide the paradigm that summarizes the relations among EIS, the electric circuit of the substance, the changes in voltage of the sensor, and the firing rate, as shown in Figure 5. In particular, it demonstrates the relation between EIS and the firing rates of FA and SA.

### 3.3. Thermal Sensation

**Figure 6:**
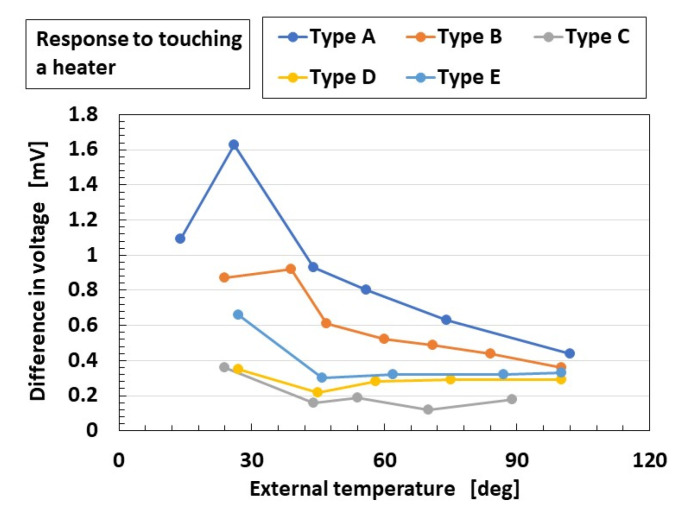
Difference in voltage in response to touching a heater.

A finger with an embedded HF rubber mechanoreceptor touching a heater with an applied external temperature is thermally responsive, which is expressed as the difference between the initial voltage and the responsive voltage, as shown in Figure 6.

**Figure 7:**
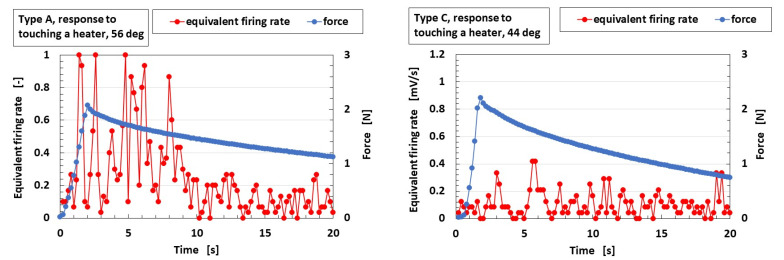
Typical cases of the relation of the equivalent rate to the application of force.

Mechanoreceptors with SA (Types A, B, and E) show larger differences in voltage, as shown at the ordinate in the figure, whose capacitance *C_P_* is larger (Figure A4 (Appendix A), corresponding to *C_2,2_* in Figure 2a). Because the capacitor is affected by heat, the thermal response is enhanced, with the result that the firing rate is faster; this tendency is also seen in the equivalent firing rate, as shown in Figure 7a. Here, in Types A–E, the equivalent firing rate is obtained from the difference in voltage standardized by the maximum voltage. As the number of larger peaks of the equivalent firing rate increases or the peaks of the equivalent firing rate become larger, the mechanoreceptor provides FA. In contrast, as the number of larger peaks of the equivalent firing rate decreases or the peaks of the equivalent firing rate become smaller, the mechanoreceptor provides SA. Therefore, Types A, B, and E provide FA, the opposite result to that obtained in the case of tactile sensation.

On the other hand, Types C and D show smaller differences in voltage, whose resistance *R_p_*, corresponding to the integrated impedance of *R_2_*, *L_2_*, and *C_2,1_* in Figure 2a, is larger. As seen in Figure A3b,d (Appendix A), the resistance *R_p_* is larger in the cases of smaller capacitance *C_P_*. Because *R_2_*, *L_2_*, and *C_2,1_* are affected by heat, the thermal response fluctuates and the difference in voltage decreases. Therefore, the firing rate decreases (Figure 7b) and Types C and D provide SA, which is also the opposite result to that obtained in the case of tactile sensation.

Thus, from the viewpoint of the firing rate, the thermal results are the opposite of the tactile results. This also means a different tendency from the real human thermal sensation. However, this tendency is related to the frequency of the response rather than to the quantitative change.

### 3.4. Gustation

**Figure 8:**
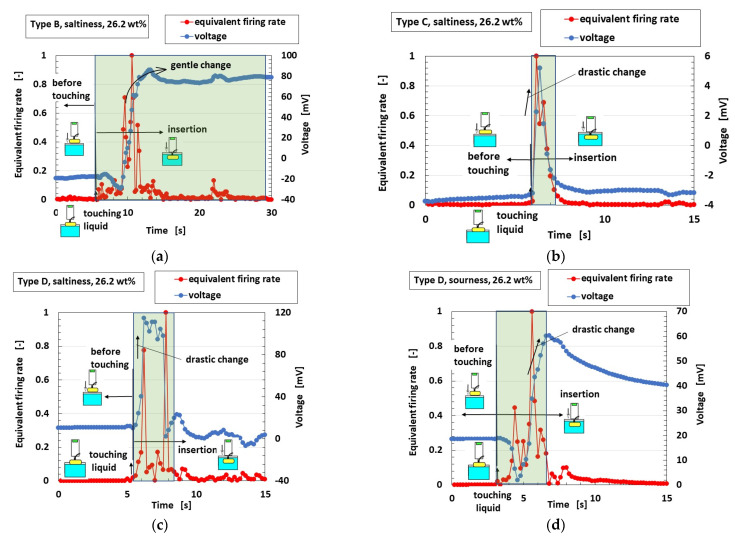

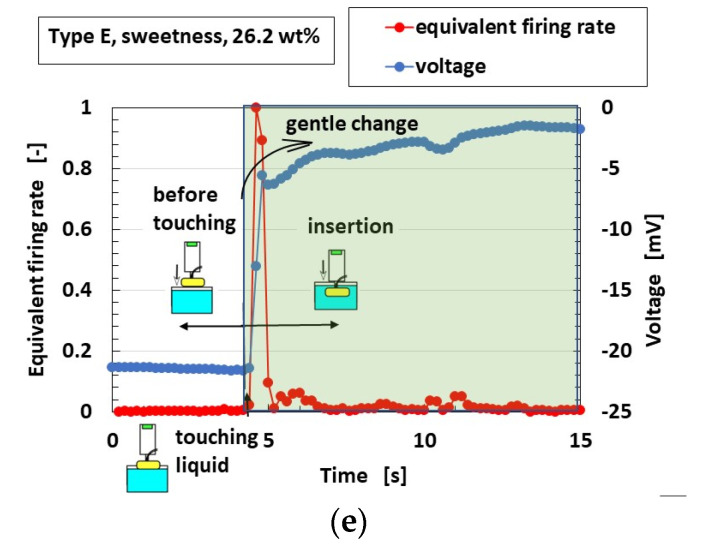
Typical cases of the equivalent firing rates and voltages related to gustatory sensation: (**a**) Type B, saltiness; (**b**) Type C, saltiness; (**c**) Type D, saltiness; (**d**) Type D, sourness; (**e**) Type E, sweetness.

**Figure 9:**
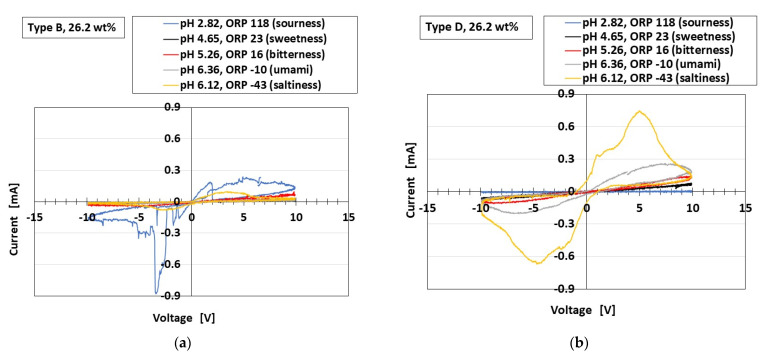
Typical results of *V*–*I* curves for gustatory sensation: (**a**) Type B; (**b**) Type D.

**Figure A5:**
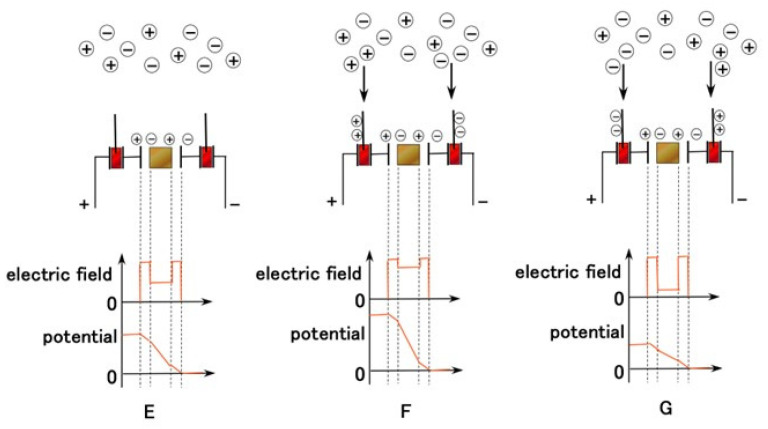
Physical model of the electric field and voltage in the equivalent electric circuits of the mechanoreceptors [34].

The equivalent firing rates calculated from the voltage and standardized by the maximum voltage for Types A–E with respect to gustatory sensation are shown in Figure 8. Type C corresponds to FAI in tactile sensation (Figure A4) and Type D corresponds to FAII, with a drastic change in voltage. On the other hand, Type B corresponds to SA and Type E corresponds to SAII, with gentle changes in voltage. Therefore, the gustatory sensation has the same adaption as the tactile sensation. This also means the same tendency as the real human gustatory sensation. In contrast, the results obtained with the potentiostat for Types B and D are shown as typical cases of SA and FA, respectively, in Figure 9. The extraneous ions of other particles and molecules of the liquids around the thin wires charge electrically to the thin wires, as shown in Figure A5 in the Appendix A [34]. Case E in the figure shows a situation in which the extraneous ions do not charge on the thin wires. In Case F, the potential evaluated by the built-in voltage between the electrodes ultimately increases in the case of thin-wire enhancement by inducement from the extraneous ions. Case G shows a situation in which the potential between the electrodes decreases in the case of the thin wires shrinking due to induction from the extraneous ions.

**Figure 10:**
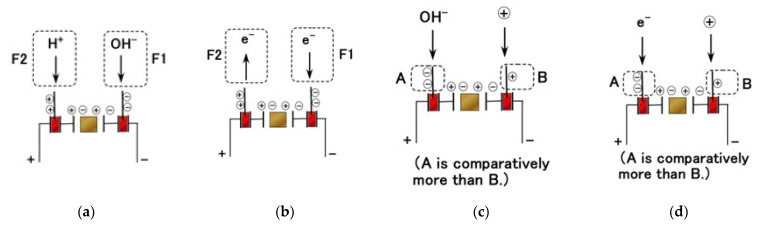
Detailed behavior of ions and electrons in the liquids in Cases F and G: (**a**) ionic transfer in Case F; (**b**) electron transfer in Case F; (**c**) ionic transfer in Case G; (**d**) electron transfer in Case G.

Case F has two types, as shown in Figure 10a, for ions entering the thin wires (H^+^ on the anode side and OH^−^ on the cathode side) and in Figure 10b for transferring electrons into and out of the thin wires. The mechanoreceptor in gustation corresponds to SA (i.e., larger *C_p_*) as shown in Figure 9a and has the behavior shown as Cases E and F. The F1 behavior (Figure 10a,b) arises in response to sourness (i.e., smaller pH and larger positive ORP), while the F2 behavior arises in response to saltiness (i.e., larger pH and larger positive ORP).

Case G has two types, as shown in Figure 10c for ions entering the thin wires and in Figure 10d for electrons entering the wires. The behavior of the mechanoreceptor in the case of gustation corresponds to FA (i.e., smaller *C_p_*), as shown in Figure 9b, and differs in pH and ORP depending on the kind of taste in question. In response to sourness (smaller pH and larger positive ORP), the response shown in either Figure 10c or Figure 10d occurs, leading to a decrease in the resistance between the electrodes corresponding to *R_p_*. The capacity corresponding to the smaller area of the *V*–*I* curve (Figure 9b) then decreases, yielding Case G. Where [H^+^] molecules outnumber [OH^−^] molecules, more OH^−^ molecules enter the thin wires on the anode side (Figure 10c). Likewise, where oxidized molecules [ox] outnumber reduced molecules [red], more electrons enter the thin wires on the anode side (Figure 10d). On the other hand, in response to saltiness (larger pH and larger negative ORP), the resistance between the electrodes corresponding to *R_p_* increases. The capacity corresponding to the larger area of the *V*–*I* curve (Figure 9b) then increases, yielding Case E or F.

### 3.5. Olfaction

**Figure 11:**
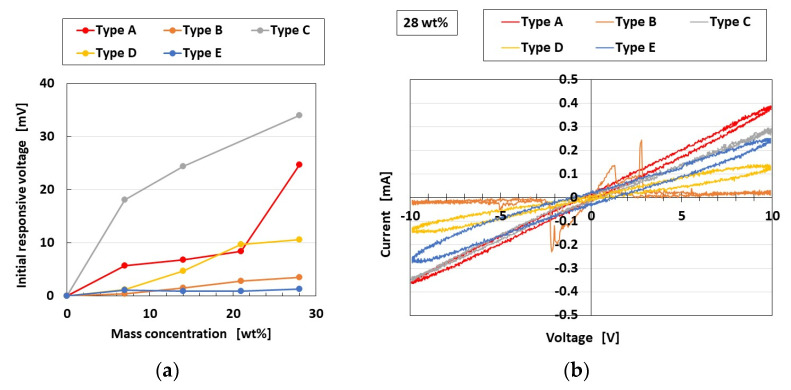
Olfactory sensation: (**a**) initial responsive voltage; (**b**) *V*–*I* curves.

**Figure 12:**
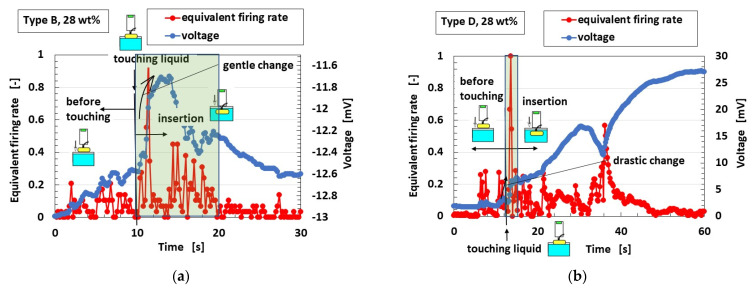
Typical cases of equivalent firing rate and voltage with respect to olfactory sensation: (**a**) Type B; (**b**) Type D.

Figure 11a shows the voltage response to an odor at the initial time of inserting the mechanoreceptor into the odor, and Figure 11b shows the *V*–*I* curves obtained with the potentiostat. Types C and D with FA show a larger initial responsive voltage and *I*-to-*V* gradient than Type B with SA. In contrast, the equivalent firing rate is calculated from the voltage and standardized by the maximum voltage for Types A–E; the typical results of Type D, with drastic changes in voltage in response to the odor corresponding to FA, and of Type B, with gentle changes to SA, are shown in Figure 12. The olfactory sensation thus shows the same adaption as the tactile sensation because the adaption is related to the behavior of ions and electrons in liquid or in air. This also means the same tendency as the real human olfactory sensation. Incidentally, the results that the gustatory and olfactory adaptions are the same depend on the following physical mechanisms. The gustatory and olfactory sensations have the similar behavior of the ions entering the HF rubber sensor.

### 3.6. Auditory Sensation

**Figure 13:**
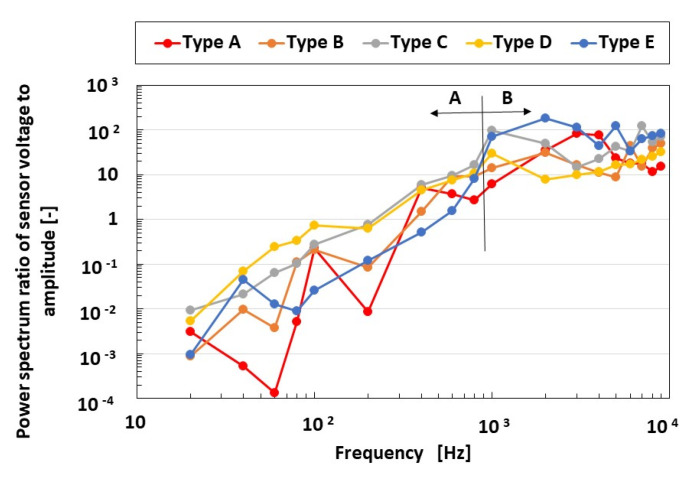
Comparison of voltage in response to the vibration of the HF rubber mechanoreceptors.

As a bio-inspired sensation, the auditory sensation is related to the response to the vibration of the cochlea in the ear. The voltage of the mechanoreceptor in response to a vibrating sound with a unique frequency applied by a speaker was also analyzed using first Fourier transform (FFT) analysis as the power spectrum, as in our previous study [7]. The analyzed parameter, expressed as the ratio of the voltage to the amplitude of the applied vibration, is shown in Figure 13. At frequencies of less than 1 kHz, the power spectrum ratios of Types C and D with FA are larger than those of Types A, B, and E with SA. In this frequency range, the auditory sensation has the same adaption as the tactile sensation. On the other hand, at frequencies greater than 1 kHz, Type E has the largest power spectrum ratio. In this frequency range, the auditory sensation is unrelated to the tactile sensation with FA or SA because the effect of the vibration of the rubber itself is dominant.

**Figure 14:**
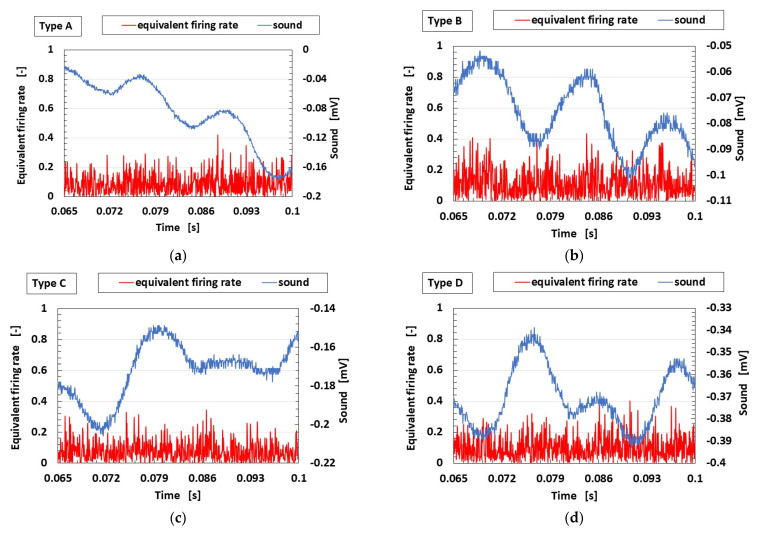

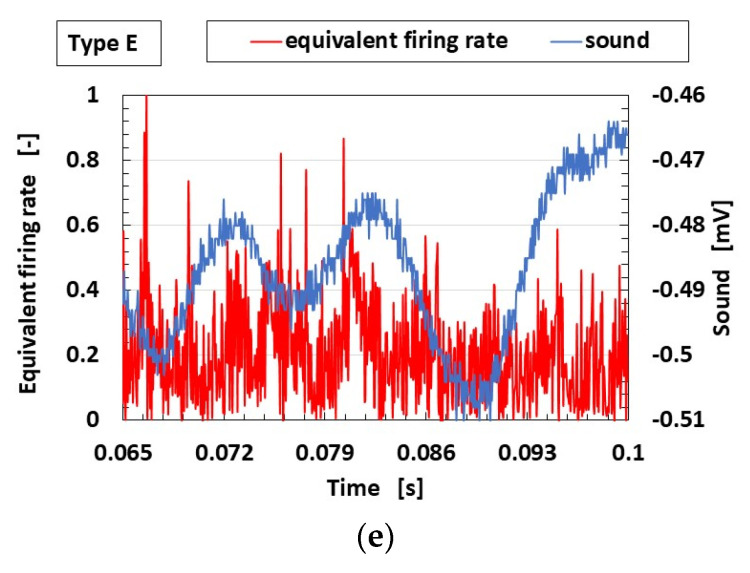
Equivalent firing rates for the application of sound for auditory sensation: (**a**) Type A; (**b**) Type B; (**c**) Type C; (**d**) Type D; (**e**) Type E.

Next, Figure 14 shows the equivalent firing rates calculated from the voltage and standardized by the maximum voltage for Types A–E. The firing rate changes in the order of E > B, D > A, C. Only Type E shows a correlation between the firing rate and the power spectrum ratio at frequencies greater than 1 kHz. Thus, at this frequency range, the auditory sensation is unrelated to the adaption of the tactile sensation because the firing rate is the evaluation of the frequency of the response and not the intensity of the sensation. This also means that the tendency in contrast with the real human auditory sensation varies according to the frequency range.

### 3.7. Consequence

Correlations among the firing rates of the tactile, thermal, gustatory, olfactory, and auditory sensations corresponding to the five senses were evaluated using EIS. EIS is a well-known method for clarifying intrinsic structures in the field of solid or ionic conductors. HF rubber mechanoreceptors have ionic conductivity, so EIS is an effective way to elucidate their firing rates for the various sensations. Impedance *L_P_*, capacitance *C_P_*, and reactance *X* are related to the firing rate. HF rubber mechanoreceptors have parallel electric circuits of units for *R_2_*, *L_2_*, and *C_2,1_*, which were evaluated as built-in currents, and for *C_2,2_*, which was evaluated as built-in voltage, with the result that HF rubber 2 may be dominant.

## 4. Conclusions

The present results are summarized as follows.

Thermal sensation: The adaption of the firing rate in thermal sensation (Types A, B, and E provide FA and Types C and D provide SA) is the opposite of that in tactile sensation. This tendency relates to the frequency of the response and is different from the quantitative multitude.

Gustatory and olfactory sensations: The firing rates in gustation and olfaction have the same adaption as in tactile sensation, whose tendency is relevant to the results of the *V*–*I* curve, because this adaption is related to the behavior of ions and electrons, whether in liquid or in air.

Auditory sensation: At frequencies of less than 1 kHz, the auditory sensation has the same adaption as the tactile sensation. On the other hand, at frequencies greater than 1 kHz, the firing rate in the auditory sensation is unrelated to the adaption of the tactile sensation.

Thus, the firing rate for the tactile sensation is different from those for the other sensations in some cases because the firing rate is evaluated according to the frequency of the sensation and not its magnitude. Therefore, if we focus on the magnitude, variant results are obtained; this is consistent with our previous results [24].

Evaluating the firing rate is significant not only in the field of neurophysiology, to research the biochemical reactions of neurons and perceptions of stimuli by the brain, but also in the field of sensors, to achieve the various sensations. In addition, HF rubber mechanoreceptors have a single-platform electric circuit so that they can be applied to the multiple sensations. The relatively simple process used in the production of these sensors has the potential to simplify the structure. HF rubber mechanoreceptors for which the firing rate can be evaluated are useful in the development of sensors mimicking bio-inspired sensations in artificial sensors. They are expected to have applications in health diagnosis, wearable human-machine interfaces, rehabilitation, etc.

## Data Availability

Not applicable.

## Appendix A

Figure A1 is a schematic of a biomedical virtual mechanoreceptor, our fabricated HF rubber mechanoreceptor, the inner structure of the fabricated electric mechanoreceptor, and the equivalent electric circuit with a single platform, as presented in our previous study [24].

Figure A2 shows the electric behavior of the particles, ions, and molecules in the HF rubber under the application of an external extraneous electric or dynamic field, as described in our previous study [7].

Figure A3 shows the results for impedance, capacitance, inductance, and resistance of the HF rubber mechanoreceptors compared with those of an ordinary electrolytic capacitor (A0830, 0.1 mF, 100 V), as presented in our previous study [24].

Figure A4 shows the relations among capacitance, inductance, and HF rubber mechanoreceptors, as presented in our previous study [24].

Figure A5 shows the behavior of electrons and ions in the test liquids for gustation and the odor for olfaction around the HF rubber mechanoreceptors [34].
